# Information-sharing experiences of professionals looking after children with cancer: a qualitative exploration from a specialist paediatric oncology unit in India

**DOI:** 10.3332/ecancer.2022.1399

**Published:** 2022-05-26

**Authors:** Trishna Chaudhuri, Devi Nandakumar, Soumitra Shankar Datta, Zakir Husain, Reghu K Sukumaran, Inder Sekhar Yadav, Sekhar Krishnan, Samiran Panda

**Affiliations:** 1 Humanities & Social Sciences, Indian Institute of Technology Kharagpur, Kharagpur, West Bengal 721302, India; 2 Department of Palliative Care and Psycho-oncology, Tata Medical Center, Major Arterial Road, New Town, Kolkata 700160, India; 3 MRC Clinical Trials Unit, Institute of Clinical Trials & Methodology, University College London, 90 High Holborn, London WC1V 6LJ, UK; 4 Department of Economics, Presidency University, College Street, College Square, Kolkata 700073, India; 5 Department of Paediatric Haemato-oncology, Tata Medical Center, Major Arterial Road, New Town, Kolkata 700160, India; 6 National AIDS Research Institute – Indian Council of Medical Research, Plot No 73, G’-Block, MIDC Rd, MIDC, Bhosari, Pune, Maharashtra 411026, India; 7 Epidemiology & Communicable Diseases Division and Scientist – G, Indian Council of Medical Research Headquarter, New Delhi 110029, India; a https://orcid.org/0000-0003-1674-5093; b https://orcid.org/0000-0002-6769-3847; c https://orcid.org/0000-0002-5077-6275

**Keywords:** children, cancer, communication, paediatric, oncology, health professionals

## Abstract

**Background:**

Childhood cancer often involves a long-term engagement of children and their parents with health services. During this journey, communications between professionals, parents and young people can be stressful for all the stakeholders. This study explores the communication preferences in paediatric oncology.

**Objectives:**

The objective of the present exploratory qualitative study was to understand the views of professionals regarding information exchange during cancer treatment of children and complement these findings with clinic-based ethnographic observation of real-life consultations.

**Methods:**

Using qualitative methods, in-depth interviews were conducted with paediatric oncology professionals. The interviews had been audio-recorded and transcribed verbatim. Alongside in-depth interviews, real-life interactions between parents, professionals and children were observed. Data were analysed using a thematic analysis framework as suggested by Braun and Clark.

**Results:**

Paediatric oncology professionals (*n* = 14) were interviewed from diverse professional backgrounds that included consultant paediatric oncologists, junior specialist trainees in paediatric oncology, paediatric oncology nurses, social workers, survivor counsellor and psychologists looking after children with cancer. Additionally, clinic-based ethnographic observations (*n* = 10) of interactions between professionals, parents and young people were also conducted. The following themes emerged from the interviews: a) Information needs of children were very different from adolescents. Children were more worried about ‘here and now’; b) adolescents were, on the other hand, mostly worried about the ‘impact of cancer on their broader life, friendships and academics’; c) parents were curious about the outcome, costs and effectiveness of treatment, and different patterns emerged for mothers and fathers; d) information needs were dynamic and different at the start of the treatment, during treatment, at remission or end of life; e) the journey of the clinicians themselves impacted information-sharing practices; and f) direct observation of consultations highlighted the importance of priming parents before delivery of information, having multiple family members during the conversation and managing intense emotions expressed during the session.

**Conclusion:**

Paediatric oncology professionals need to be sensitive about the dynamic nature of information needs while interacting with children and parents of children with cancer. The above findings may help tailor the discussions that professionals ought to have with families with a child with cancer. The results may contribute to the understanding as well as to developing training courses on communications in paediatric oncology for low- and middle-income countries.

## Introduction

In 2020, there were 279,419 children and adolescents globally, aged 0–19 years, with newly diagnosed cancer [1]. About 15% (14.65%, 40,924/279,419) of such patients were from India [1]. Over time, the treatment outcome of childhood cancers has improved significantly in high-income countries. However, this is not the case in many low- and middle-income countries (LMIC) due to the scarcity of dedicated paediatric oncology treatment facilities, lack of financial support, and logistical problems leading to treatment abandonment [2]. Treatment abandonment in childhood cancers is a common problem for Africa and India [3, 4]. There are reports from Ghana that the quality of information shared with parents of children with cancer influenced the retention of children within cancer treatment programmes [5]. Information exchange, parent education and developmentally appropriate communications with children with cancer are recommended in high-income countries [6] and LMICs [7]. Effective communication helps to engage the child, addresses unknown fears, improves treatment adherence and contributes to better symptoms management [8]. A recent review from the USA concluded that clear empathic communication between paediatric oncologists, children and their parents fosters better therapeutic alliance and aligns medical management to the expectations of the child and the family [6].

Breaking bad news is never easy and is fraught with challenges for parents and professionals [9]. Research from the Netherlands has shown that young people with cancer, cancer survivors and parents prefer to be fully informed about cancer care [10]. The above-mentioned studies also highlighted the importance of interpersonal relationships between patients and professional care providers as communication in paediatric oncology may be emotive and may often deal with the uncertainty of the outcome.

Medical information and communication systems likely deal with medical constructs that have non-linear relationships with several other parameters and are likely to be less predictable, despite robust measurement methods for some of the values [11]. Repeated observations, interpretation of complex interdependent values and adjustments in position based on the patient’s health, and prognosis impact communication heavily [12]. This can create tension during information exchange between the professionals, patients and their caregivers in oncology [13].

Hospitals are under constant pressure to provide a level of care that reaches the family’s expectations. It is essential to listen to the views of the patients and their families and be ready to adapt the services over time. The specific interaction pattern of any particular clinician depends on several factors, like past experiences of that person with similar patients, skills in handling difficult conversations, and various conscious and unconscious factors [14]. Over the past two decades, one of the critical areas of change has been a move towards open and transparent communication between the service users and the clinicians.

Using a qualitative design, the present study explored the communication and information exchange between professionals and children with cancer and their parents in a specialist oncology setting situated in an LMIC. The study also investigated the emotional impact of delivering such information on the professionals before, during and after the news is communicated. The interview-based data are complemented by findings from actual observations of information exchanges captured in outpatient clinics, paediatric oncology wards, day care and hospital emergency departments.

## Methods

### Design

The present study followed a cross-sectional design using in-depth qualitative interviews of professionals managing children with cancer complemented by observations using clinic-based ethnography by a sociologist. Healthcare workers from different professional backgrounds, such as doctors, nursing staff, social workers, survivor counsellors and paediatric nutritionists, were included in the study. The study was approved by the institutional ethics committee (2018/TMC/131/IRB27).

### Setting

The study was conducted in the paediatric oncology department of a large tertiary cancer hospital catering to a wide geographical catchment area in eastern India and its neighbouring countries Bangladesh, Nepal and Bhutan. The paediatric oncology department treats all types of children’s cancer for patients up to 18 years of age and has several junior and senior paediatric oncologists, dedicated wards for children and adolescents with cancer and an around-the-clock multidisciplinary clinical service. The study hospital is a non-profit charity and offers subsidised treatment to patients, many of whom are children. As a result, the children and adolescents seeking cancer treatment are from a wide socio-economic background.

## Participants

The study recruited professionals involved in cancer care to explore their perceptions on information sharing and care plan discussions. Potential participants were approached by the researchers and written informed consent was obtained before recruitment to the study. Purposive sampling strategy was followed, keeping in mind to include a wide range of respondents, in line with the principles of standard qualitative research. Additionally, observation of actual information sharing was conducted during the real-life discussion in outpatient clinics, paediatric oncology wards, hospital emergency and day care settings.

## Study team

The study team consisted of a consultant psychiatrist, a clinical psychologist, a sociologist, two consultant paediatric oncologists, a public health specialist and two academic health economists with special interest in public health. Two of the senior members of the team had considerable expertise in conducting and leading qualitative research projects. The two research interviewers consisted of a clinical psychologist and a sociologist, both of whom were trained in the techniques of qualitative interviewing, observation methods and data analysis by the senior researchers. They had the required experience of handling difficult conversations and were thus suited for this project that was bound to bring up a variety of emotional topics during discussions. The two interviewers were not part of the paediatric oncology team from which the professionals were recruited. As a result, the interviewees could freely discuss any topic that they felt was relevant to the research area.

### Data collection

#### Interviews

The interviews were conducted in a private office at a convenient time chosen by the respondent. There was no one else other than the participant and the interviewer during the interview. The interviews of the participants included asking about socio-demographic details, followed by an in-depth interview [15] exploring the topics of interest using an interview guide. The initial interview guide was made by a consultant paediatric liaison psychiatrist (SSD) and a clinical psychologist, and questions were added based on the subsequent interviews, including topics that felt important to be further explored. Open-ended questions probed areas like communication preferences with children with cancer, differences between children and adolescents, preferred style of conversation and impact of such conversation on the clinicians and patients, and related topics. All the interviews were audio-recorded and transcribed verbatim. To begin with, the interviewees established a rapport with the participants by asking a few neutral questions, followed by a few open-ended questions and further questions that clarified the specific answers when required. Data collection and data analysis took place simultaneously. The interviewers also maintained field notes and personal reflections that they made during or just after the interview.

#### Clinic-based observation

The semi-structured clinic-based ethnographic observation method [16] was used to observe clinicians’ interactions with sevice users in real-life settings. Clinic-based ethnography has been used earlier to study clinical interactions [17]. The ethnographic observations consisted of maintaining notes on the interaction observed and reflection notes made by the researcher. Information exchange in paediatric oncology can be sensitive and may generate intense emotions, especially at the time of the initial diagnosis, during relapse and at the end of life. Emotional experiences can be valuable and often essential sources of information in ethnography [18]. The researchers also made notes on the emotions generated during and following the clinical interaction.

#### Data analysis

The data generated from the in-depth interview were analysed using the framework of thematic analysis. N-Vivo software was used for the qualitative data analysis and storage. The interviews were coded by two researchers. A third researcher reviewed all the codes and helped to sort out any differences. The sequential steps of qualitative data analysis were (a) generating codes, (b) charting the data, (c) data synthesis, (d) generating basic themes and (e) generating global themes as described by Braun and Clarke [19]. The field notes and reflection notes of the interviewers and researchers were also transcribed and analysed to complement the findings of the interviews.

#### Results

Participants recruited for the study represented a wide range of professionals working in the field of paediatric oncology. Sixty-four per cent (9/14) of those interviewed were women, reflective of the usual gender mix of multidisciplinary paediatric oncology teams. The study included professionals who had a work experience of 1–20 years in the field of paediatric oncology. The direct observations were carried out in outpatient clinics, inpatient wards, day care settings and emergency departments. The respondents’ characteristics are presented in Table 1.

**Figure 1.**
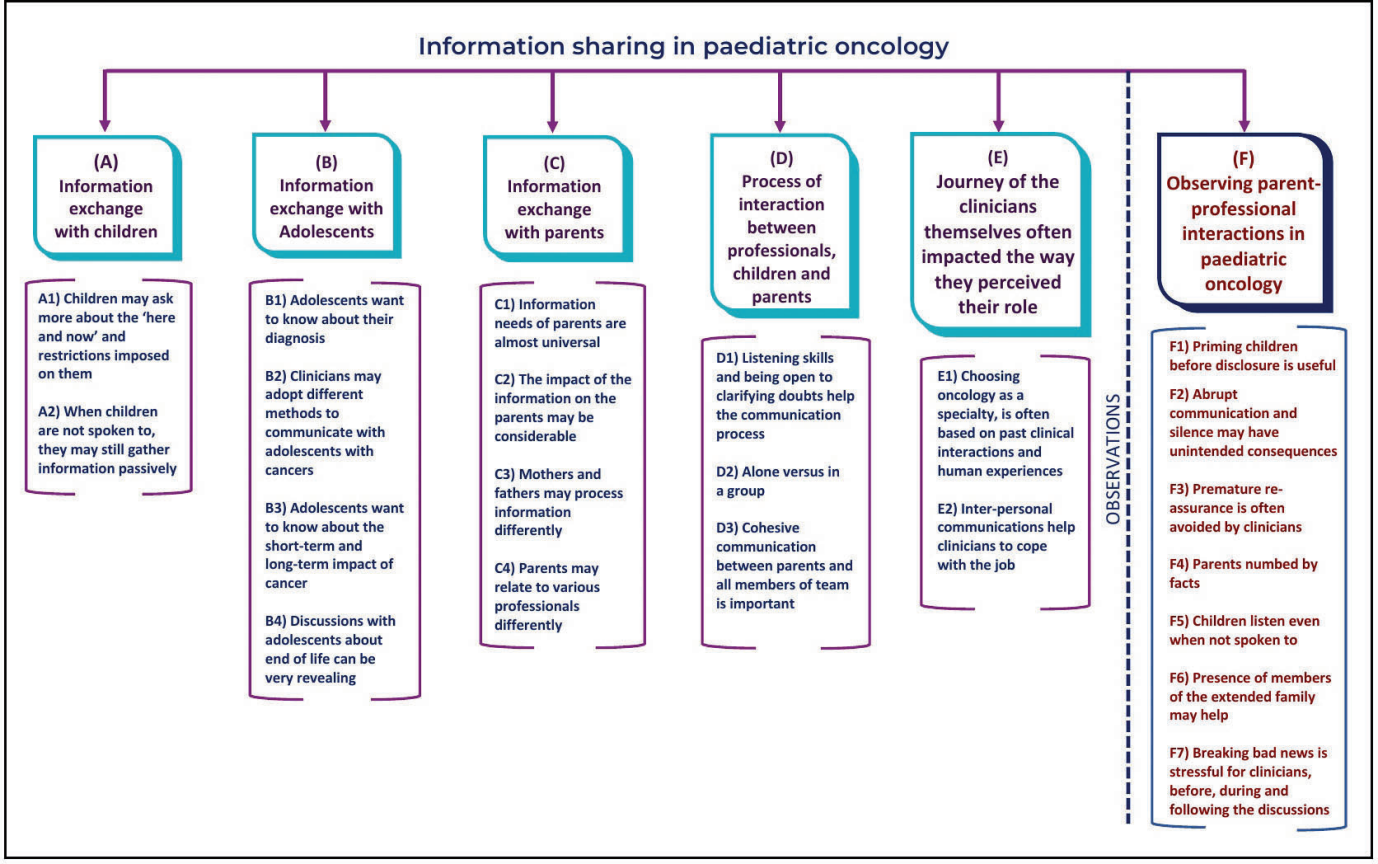
Coding tree: Information sharing in paediatric oncology.

The interviews and observation elicited various themes that are shown in Figure 1. Many of the views expressed by the clinicians were confirmed in the actual observations included in the study. This section will first describe the themes elicited from the in-depth interviews and following this, observation findings will be reported.

### A) Information exchange with children

#### A1) Children may ask more about the ‘here and now’ and restrictions imposed on them

On asking professionals, about the information needs of very young children, some of the professionals said that they did not have significant information needs.

*‘Very young kids, meaning those who are under five years, don’t understand anything anyway. They are only scared of seeing things. They may be scared of nurses and doctors’. (P11, Survivor Counsellor)*

Professionals shared that at the start of the treatment most children did not know what cancer was. They often asked why they were in the hospital.

*‘Children don’t know what is the meaning of cancer. They may ask why are they here. Why are they not in the other hospital near their home? Why do they need to stay away from home for such a long time?’ (P1, Doctor)*

Another professional felt that some children, below the age of 12 years, might have a degree of familiarity with a few of the commonly used medical terms without understanding the full meaning of these words.

*‘If the child is very intelligent, they understand a little bit. They may say, ‘Will have chemo’, ‘I don’t want IT’. But they may not understand the factual details or the seriousness of the situation. Cancer can be just like fever to them. Just like a simple thing’. (P6, Doctor)*

#### A2) When children are not spoken to, they may gather information passively

One of the respondents said that at times he had been surprised by a child’s degree of understanding.

*‘You may not be concentrating on the child. You are possibly talking to the mother or father at the foot end of the bed and completely ignoring the child, and that is when they suddenly come up with something that makes it obvious that they already knew’. (P3, Doctor)*

Another clinician clearly said that if children do not have their condition explained to them, they may misunderstand things.

*‘They start thinking that something bad has happened. Maybe they won’t get well and get back home. They ask these questions to their parents with lots of anticipation’. (P11, Survivor Counsellor)*

*‘I found when they overhear many things, they may get a misconception about things’. (P9, Counsellor)*

### B) Information exchange with adolescents

#### B1) Most adolescents want to know about their diagnosis

While discussing cancer-related information, clinicians differentiated children from adolescent cancer patients. Many of them believed that adolescents need to know about the disease and its treatment. One of the doctors discussed the moral dilemma that clinicians go through before breaking bad news to adolescents.

*‘Adolescents, I think, are a bit different. They need to be told. And as a clinician the dilemma is that, should I tell him or should I not tell him, should I protect him or not protect him. How they are going to cope? How are they going to take this information?’ (P3, Doctor)*

A clinician expressed her opinion that adolescent cancer patients value frank discussion with clinicians.

*‘I think if it is an older child, his or her major concern is about the outcome of the disease. They want an honest answer, as much as you can offer them. They want you to be as truthful as you can’. (P7, Doctor)*

Some adolescents may not ask questions directly but will try to gather information indirectly.

*‘They do not want to ask any question directly. When we go for ward-rounds and talk to the parents they will try to figure out [voice cracked]. You know that they are listening, they are trying to catch on to every single word that you are saying’.(P2, Doctor)*

#### B2) Clinicians may adopt different methods to communicate with adolescents with cancers

There may be variations in the way adolescents engage with clinicians. Respondents said some of them were reserved, while others opened up easily. They emphasised that clinical engagement with an adolescent is also not a static process and evolves. Adolescents bond with the clinicians better as the treatment progresses.

*‘I feel that adolescents are difficult to manage. Meaning, they are difficult to predict. You will see some of them may not open up throughout the treatment. But many of them will be interactive, they will befriend you. And in this age of the internet, they will catch you on the internet as well. But some of them would not talk to you as much. You cannot often predict who is going to behave in which way. When the duration of treatment is longer, the more they see you, the more they interact with you. And then the relationship eases a bit’. (P6, Doctor)*

Clinicians used developmentally appropriate language while discussing treatment details.

*‘Kids who are 12 years and above, are informed (about the diagnosis). They are informed that they have a blood-related disease that needs some treatment, and this will result in them not being able to go to school and resulting in their hair loss. Since these kids are a bit matured, they understand’. (P11, Survivor Counsellor)*

*‘For surgeries done to remove bone tumours, today there are options of prosthetics. So, if we know that a major chunk of the bone is going to get removed, we tell them’. (P7, Doctor)*

#### B3) Adolescents want to know about the short-term and long-term impact of cancer

After getting to know about their cancer diagnosis, adolescents often ask questions related to the potential future impact of the treatment.

*‘For adolescents, the first question they ask is about ‘missing schooling’. Many of them may be appearing for the boards if they’re in higher classes. They ask ‘Is there going to be a gap?, Are they going to miss a year or more than that? They are worried about that’. (P7, Doctor)*

*‘They are going to school or college. Suddenly they are out of the college, they do not meet with their friends, they are angry because they are not able to do their routine activities and they have to be in hospital most of the time’. (P6, Doctor)*

There may be differences in their reactions to the cancer diagnosis, depending on the gender of the young person.

*‘Boys can get very angry. “Why me?” I have plenty of things to do. I was dreaming so much (of the future). I was supposed to go to college but why is this happening to me? So they can become angry and agitated. But girls usually take it very very calmly’. (P3, Doctor)*

Clinicians went on to elaborate that the second common concern that adolescent cancer patients mentioned was about changes in their appearance. Several respondents said that this was true for both genders but more frequent with adolescent girls than boys. The usual questions revolved around loss of hair or skin changes due to the treatment.

*‘They are [takes a deep breath] extremely worried about hair loss, with a line sticking out, and NG tube sticking out. I think they are worried and this I think can happen for both genders. But I think that the girls are way more conscious of this (their appearance). And a good number of times they have initially refused a particular treatment because of these concerns’. (P2, Doctor)*

*‘A second concern is about the looks, the peer pressure you know, and especially girls are upset that there’s going to be alopecia’. (P7, Doctor)*

Adolescent patients also have concerns about skin changes.

*‘If she starts on some therapy, that is associated with skin changes, like darkening of the skin or lightening of the skin caused by drugs like Imatinib, they may have questions about that. They may ask if the skin will darken again when you stop the medicine’. (P7, Doctor)*

One patient who underwent amputation was not comfortable sharing about it with his peers. He was particularly sensitive to any fellow patient seeing the bare stump of the amputated leg without the dressing on.

*‘He was 15 years old and uncomfortable showing his amputated leg to other people. A surgeon was dressing his wounds in the post-operative period without the curtains on (around his bed) and he just cried a LOT after the doctor left’. (P8, Social Worker)*

Some patients anticipate relationship problems and suffer due to this after learning about their diagnosis.

*‘One adolescent girl knew that her boyfriend will leave her when she was diagnosed. She was distressed for the last 3 months of her life and suffered a lot’. (P8, Social Worker)*

One doctor recalled that she had interacted with a few adolescent boys with testicular relapse of acute lymphocytic leukaemia who were worried about their future reproductive abilities.

*‘Specifically, the boys with testicular relapses asked whether they will be able to father children. In a span of one month, three adolescent boys I have seen asked me this question’. (P1, Doctor)*

Even after recovery, adolescent cancer survivors may be worried about the recurrence of the disease.

*‘He had a rash on his face. Like pimples. And he also had a fever for two days. He thought that he had relapsed. He was very distressed. So, he came to the hospital. Then, CBC (Complete blood count) was done, and counts were okay. He had some rash and pimples. So, the doctor gave him some medicines. And now he is all right. So sometimes with any fever or nodule, they think that their disease is coming back’. (P8, Social Worker)*

#### B4) Discussions with adolescents about end of life

In the unfortunate situation of a young person facing the end of life, some of the older children demonstrated a mature level of understanding. One of the clinicians recalled the statement of a dying child with leukaemia to his mother. His disease had relapsed after bone marrow transplantation.

*‘One day he told his mother “Now free me from this. I want to go”’. (P10, Nurse)*

This sentiment of dying adolescent cancer patients understanding the gravity of the situation was also echoed by other clinicians.

*‘If it is an adolescent, they know they are going to die. They may not be talking about it to their families, and their friends. And they may even feel lonely and think they are alone in this world’. (P8, Social Worker)*

### C) Information exchange with parents

#### C1) Information needs of parents is almost universal

Parents always want to know about their children. On asking a professional about parental preferences for gaining information regarding their child’s cancer, he emphasised that ‘all’ parents want information.

*‘Everybody wants to know. No exception. Everybody wants to know every single bit of information. That is for sure. How much they will understand, how much they will take in, how much they will imbibe, how they will synthesise, what action they are going to take, whether I am going to influence their decision making - all that I do not know. BUT, everybody wants to know’. (P3, Doctor)*

There may be a difference in the amount of information that parents want to know, depending on their level of understanding and educational background.

*‘Those who are educated want to know many things. They will ask many questions. Sometimes they will ask things that even doctors don’t know. They will come with materials they have come across on the internet, based on the diagnoses and the investigation results. They will ask why can you not do all those things with their child. There are other parents who don’t understand so much. These parents may not ask many questions. For them, you have to give all the information. Both extremes exist’. (P10, Nurse)*

#### C2) Impact of the information on the parents may be considerable

Parents may react to cancer-related information differently. They may also filter and take in ‘what they want to hear’.

*‘The majority of the people do not want to hear what they do not want to hear’. (P3, Doctor)*

Most parents feel very upset about their child’s cancer. Some blame themselves.

*‘Did I do something wrong? Or should I have or not have done something which could have prevented this for my child? Am I too late?’ (P3, Doctor)*

Some parents express guilt and hold themselves responsible for their child’s cancer coming back.

*‘Oh I should not have given him the ice cream, I should not have taken him out outside. I should not have sent him back to school’. (P3, Doctor)*

Accepting to move over to palliative treatment is difficult for any parent. Sometimes parents will not believe the doctor. But this position may change over time.

*‘We know that they will come around, and then a good number of times the disbelief ultimately turns into belief. You may have to go around in circles [takes a deep breath]. But some parents, they would not take that “No” for an answer’. (P2, Doctor)*

However, there are parents for whom the transition can remain very difficult. They may be verging on ‘disbelief’ and often ‘blaming’ the treating doctors.

*‘We have been called names, for sharing bad news. One parent went on to say that “you shouldn’t be treating children if you are giving this kind of treatment” and started desperately negotiating “How can you tell this to a parent?”’ (P2, Doctor)*

#### C3) Fathers and mothers may process information differently

The role each parent takes up in the caregiving process may have a pattern.

*‘Regarding finances, fathers mostly deal with it. Mothers mostly are always there with the children’. (P11, Survivor Counsellor)*

*‘I think during the initial period (after diagnosis) mothers are always allowed to vent out. I think the father has to be the protector. So he never cries. Never shows emotions’. (P1, Doctor)*

As time goes on, mothers get used to the child’s cancer treatment as they are with the child most of the time. If the clinical condition of the child deteriorates, it is almost always in front of the mother. Fathers may not be around so much and, as a result, may have lesser time to prepare psychologically for any clinical deterioration. For this reason, fathers may lose all hope abruptly.

*‘In the ICU, with all the tubes in, that is the point you know when “he” (father) gives up’. (P1, Doctor)*

#### C4) Parents may relate to various professionals differently

A senior nurse, working in a paediatric day care facility, commented that parents often ask more intimate questions to nurses as they feel comfortable discussing these issues with them.

*‘I look after patients in the day care. When they’re coming to the day care, they have been already counselled by the doctors and their treatment has started as “inpatient”. Sometimes they may ask if they are okay to have another baby’. (P10, Nurse)*

A survivor counsellor working alongside doctors and nurses spoke about her unique childhood cancer survivor perspective. She felt that the parents of children with cancer felt hopeful after they spoke to her.

*‘Earlier she (names the mother of a child) used to see me working in the ward. But when she got to know that I too had a similar illness, she came to me and asked me everything in detail. When I explained my condition to her, she told me “I was scared earlier. I did not know if my daughter will be able to do anything after she gets well. But looking at you, now I feel hopeful that, maybe, someday, my daughter will also work somewhere”’. (P11, Survivor Counsellor)*

### D) Process of interaction between professionals, children and parents

#### D1) Listening skills and being open to clarifying doubts help the communication between clinicians and service users

One professional highlighted the importance of letting parents ask questions and clear their doubts.

*‘I can go to the parents and give a lecture. That is never effective. I let them talk, ask questions and clear their doubts. And then address those issues during the discussion. Whatever doubts they have, questions they have, I try to answer those questions. That way they will understand what is going on. It is about continuous communication, and effective communication, and avoiding miscommunications’. (P4, Psychologist)*

She went on to elaborate that while breaking bad news, the ambience, the manner of doing it and other factors are very important:

*‘While breaking bad news, the room, the ambience of the room, the environment, all are important. The room should be quiet. The way you sit in front of the parents, your appearance, your way of talking, at what point of the discussion you deliver that news - all that matters. Depending on the way you deliver the news, the reaction may be different’. (P4, Psychologist)*

Another professional spoke about how his focus on communication has changed over the years. His focus is now on understanding the family’s point of view and is not limited to sharing cancer-related information.

*‘Previously I was not mature to handle these conversations. Now I start with trying to understand the family’s point of view. Why they are protective? I try to look at it from their point of view’. (P10, Nurse)*

#### D2) Alone versus in a group

One professional highlighted the differences in the interaction, depending on who is present at the time of the discussion.

*‘When they are alone they generally ask about their fears. “Will I get well?” “Will I be able to go back home ?” These are the questions they ask when they are alone. When they are in a group they may behave differently. They may say “we went to meet Sourav Ganguly (former cricket captain of India)” or they make song requests one after the other. “Please play that (names a specific song) song ma’am”’. (P11, Survivor Counsellor)*

#### D3) Cohesive communication between parents and all members of the team is important

Professionals have understood that parents are likely to clarify the messages shared with them with different members of the team and highlight the importance of having a cohesive narrative.

*‘Communication to the family members from every single level should be the same, whether it is the nursing staff, whether it’s the junior doctors or whether it’s the consultants. Everybody should say the same thing’. (P3, Doctor)*

### E) Journey of the clinicians themselves often impact the way they perceived their role

#### E1) Choosing oncology as a specialty is often based on past clinical interactions and human experiences

Clinicians, who chose to train and work in paediatric oncology, had their own stories to share. These stories highlighted the reasons behind selecting the subspecialty and their reflections on working with children with cancer. They often cited specific interactions with parents and children that had motivated them to choose their field of work. One of the doctors mentioned the reason behind her choosing to train in paediatric oncology was to do with her feeling helpless and emotionally moved to see children die of cancer while working in a hospital near her home, with no infrastructure to treat children with cancer. She had travelled a long distance to complete a fellowship in paediatric oncology. She plans to train and return to the same place to start treating children with cancer more effectively. Some of the junior trainees had travelled across international borders to train in paediatric oncology.

*‘I have done my graduation in XXX (name of a city). I have also done my specialisation (in paediatrics) from the same place. And I know oncology is struggling there. So many kids died in front of my eyes’. (P1, Doctor)*

*‘I am from Nepal. In Nepal, paediatric oncology, as a sub-specialty, is still in its nascent phase. There are very few paediatric oncologists working there (in Nepal). So you often see patients traveling to India to get the treatment in reputed paediatric oncology centres’. (P6, Doctor)*

A survivor counsellor spoke of her ‘lived experience of a cancer patient’ to be the most crucial deciding factor.

*‘I thought if I ever do something, I will do something with cancer patients. That became the goal of my life since then (after completing my cancer treatment). So when my treatment was completed I got introduced to the professionals from Cankids. Then I joined XXXX while continuing my graduation’. (P11, Survivor Counsellor)*

#### E2) Interpersonal communications help clinicians to cope with the job

Working in the field of paediatric oncology was reported to be fulfilling, but at the same time challenging. One professional believed that the practice of medicine is based on science. He tried to use this belief in protecting him emotionally.

*‘You do whatever has been taught to you, in your practice. This is based on scientific principles. The result is not in your hand. That’s all you have to believe’. (P3, Doctor)*

However, many professionals spoke about feeling upset when a child died.

*‘It’s very difficult. It was very difficult for me initially’. (P5, Nurse)*

*‘Sometimes I can’t sleep, I can’t do anything when a child expires or the disease relapses. It bothers me [she gets emotional, looks down and tries to control her emotions]’ (P10, Nurse)*

It becomes challenging if one is closely involved in caring for a particular child who dies in front of them. The perception of being close to a patient was based on the clinician’s interactions with that specific child.

*‘She underwent a transplant. She was quite close to me. Wherever she would see me, she would come. But sadly, she passed away on the day I was on duty. That was very bad. I could not do anything. It took me a few days (pause). It affected me a lot. Because it made me so emotional and there was none to support me. Even at home, there was nobody there on that day. So, later on, I talked to one of my seniors’. (P6, Doctor)*

She went on to elaborate the importance of this inter-professional communication and support as follows:

*‘We go through this emotional turmoil when you have cared for a patient for quite a long time. Then suddenly on night duty, he collapses and dies. That becomes an emotional burden. To relieve this pressure one needs to do something. As this ultimately has a bearing on the work. So, the colleagues or those working with you, have to talk to you. They are the ones who can help you the most’. (P6, Doctor)*

Some professionals personally identified the sufferings of the children in the hospital with their children at home. One clinician said that she spoke to one of her colleagues about her emotional distress.

*‘I have a 7-year-old girl. So, when I joined here she was only 4 months. At times, it can be emotionally challenging for me to care for children with cancer. Some days I come to XXXX to talk about my pent-up emotions’. (P10, Nurse)*

Even after working many years in the sub-specialty, a clinician became emotional when asked about ways of coping with the job.

*‘You just said what I have been doing for so many years. And how many pieces of my heart have I lost? Every time…. [takes a deep breath]. Just because I am a senior consultant, people just think that I will absorb every emotion effectively. But who comes and asks me how am I doing? And it was SUCH an eye-opening moment for me, YES, nobody comes and asks me’. (P2, Doctor)*

Some clinicians found their families to be a significant source of emotional support.

*‘I would usually share these emotions with my family. I have had lots of support from them’. (P2, Doctor)*

A rare point of view was from a professional who always felt very much in control of his/her own emotions.

*‘I am very good at explaining everything to myself, how to handle and protect myself and my family during the hardest of times. That entire thing comes from within me’. (P11, Survivor counsellor)*

One doctor reflected that people develop their ways of coping.

*‘You develop your own system over time. Some listen to music, some go for a swim, someone may go out cycling. Others may be read books. You will develop a mechanism of your own for coping with all the stress and the deaths and unexpected results so that it doesn’t affect you personally. You have a life beyond your workplace. Your work-related stress and emotions should not affect your family members and friends. That is not fair to them’. (P3, Doctor)*

### Observing professional communications in a children’s cancer unit

To complement the in-depth interviews with cancer clinicians, we observed actual interviews and information-sharing practices between children, parents and their families across different parts of the hospital. The observations generated the themes explained below.

#### Observation Theme 1: Priming children about the situation without going into all the details may help in engaging the child

Children with cancer either presented to the outpatient department of paediatric oncology or to the hospital emergency. Clinicians often took time to share all the information. During the first consultation, they often informed the children of what they needed to do in the next few days. During this discussion, clinicians often did not explicitly mention the word ‘cancer’, even if they had high suspicion for the disease. Over time, clinicians engaged the child more and more in their treatment-related discussions. Children’s haematological cancer often requires the child to be admitted in the first few weeks. The scope of discussions was broadened during the admission depending on what questions the child asked and what the clinician perceived the child needed to know. This approach was often felt appropriate by the child’s family as well as the oncology team (Box A).

Box A **Milieu**: During the first outpatient clinic appointment with children’s cancer services, a child’s father requested the doctor not to mention the diagnosis to the child. They were worried that if told he might not be able to handle it emotionally.

**Doctor–patient interaction:** When the boy came for a check-up, the doctor built a rapport and did not use the word 'cancer'. She used the word 'leukaemia' instead.

**Impact on the patient:** He broke down in between and throughout the session was seen to be shivering.

**Question by the researcher to the doctor after the patient and family had left the room:** ‘Isn't it true that he will anyway come to know? He is an adolescent and he knows the word leukemia. He also knows that he has come to Tata Medical Centre, which is a cancer hospital’.

**Dr. X replied:** ‘He would know it very soon. But right now, if we had told him everything then he might be unable to handle it. He is not prepared mentally. So, let it be. Now he will go back home, he will search on the Internet, and some doubt will remain. He will be a bit more prepared for the discussion when he is back. After he is admitted, we will tell him everything’.

One observation captured the process of priming of parents regarding the recurrence of the disease over two sessions on the same day (Box B). The process of slowing the pace of conversation was also observed when discussing recurrence or during the transition from curative to palliative care. During one observation, it was seen that the doctors shared some of the information in the morning and then met the parents again on the same day to complete the conversation. This allowed them time to get ready for the bad news.

Box B **Morning:**

The father of the child was primed in the morning. The reports were reviewed jointly by the clinician and the father. He was told how the blood counts have again dropped, and how the other reports were also beyond the normal range. He was informed that the doctors will discuss these in detail in the afternoon. In the afternoon full disclosure was done.

**Afternoon:**

*Doctor to father:* ‘Why do you think we have called you?’

*Father (After a brief pause)*: ‘Maybe something is not right’. (Pause)

*Doctor:* ‘*Hmm… yes. We have called you to share very bad news. His cancer has come back and it is in a very advanced stage’.* (pause)

*Father:* ‘*Hmm, we thought so. He was not doing well recently. What should we do now?*’

*Doctor:* ‘*We are afraid that maybe we won't be able to save your child*. (Pause). *But we will focus on keeping him comfortable*.’

Father: ‘*Is there nothing that can be done?*"

Doctor: ‘*No. And some of the treatments that are available may not be suitable for him*’.

The father was in tears. The doctors gave him time to process the news. One of the fellows patted on his back to provide him comfort. After a while, the father collected himself and asked if the child could be kept pain-free until the end.

#### Observation theme 2: Abrupt communication and silence can have unintended consequences

In one of the observations, it was noted that the sudden delivery of information disengaged the child’s mother from the service providers. The mother was informed about the severe nature of the health problem. She seemed stunned and following this she stopped participating in the discussions with the clinicians and often avoided eye contact. She remained so even in the subsequent outpatient clinic visits.

On another occasion, it was observed that the doctors had remained silent after the mother of the child had enquired about other treatment options when she was informed about moving the goals of care from curative to palliative. This silence gave a feeling of an unfinished conversation. The parents spoke less and tried consoling each other through physical touch. They stopped questioning and left the room (Box C).

Box C The family was given the news that the child's treatment goals will move from curative treatment to palliative treatment. After hearing this, the mother was heartbroken and wanted to ask about alternative options, chances of recovery and what caused such a change in the treatment plan. The doctors empathized with the distress of the mother. The conversation seemed to have come to an abrupt halt. Father patted on the mother’s back and asked her to be quiet and left the OPD.

#### Observation 3: Premature reassurance is often avoided by clinicians

Parents are often keen to know the prognosis and stage of the disease at presentation even before the diagnostic evaluation is complete. Clinicians consciously avoided providing premature reassurance to parents. Sometimes very early improvement could be attributed to natural fluctuations of symptoms in any disease. At other times, transient improvement of the symptoms could be in the setting of an otherwise grave situation. Even during the treatment, the clinicians were mindful that it is not unusual for parents to misinterpret small improvements as significant progress. Being optimistic during the discussion was balanced with the need to remain grounded in the reality of managing a difficult illness (Boxes D and E).

Box D **Avoiding premature reassurance at diagnosis**

The family was very anxious to know about the outcome of the disease even before the evaluation was completed.

*Doctor:* ‘Mother, we cannot say anything until the reports are ready. Til then we cannot tell you what will happen and what will not happen. You have to be patient. The reports will be ready by next week. We will then be able to tell you what the situation is and exactly what you have to do with some surety’.

The mother seemed to be a bit upset and said, ‘*All of you have so many patients to treat. We have only one. We just need one ray of hope to sail through this difficult time. That’s all we can say*’.

Then the doctor said, ‘We understand your situation. But right now, all we can say is, you will have to wait until the reports reach us’.

Box E ‘The news of remission might seem comforting right now. But I would like to reiterate that we can soon be in trouble. It is just temporary remission. If she continues to be fine, nobody will be happier than us. But let’s wait before we conclude. We will have to run a few more tests in the upcoming months. Only after looking at those reports, we can say what the situation is and what needs to be done’.

*Mother:* Hmm. I understand.

Another observation was regarding a child who had a disease with an extremely poor prognosis, who showed some signs of improvement in the interim blood parameters. Although the parents wanted to interpret this as a sign of recovery, the doctors were candid in their conversations to not raise the family’s expectations and jeopardise the grieving process. The family had been earlier informed about the overall poor prognosis of the illness.

#### Observation 4: Parents may be numbed by facts

Communication with families of dying children is difficult for both the clinicians and the family members. In most of these sessions with the parents, doctors are under pressure to communicate honestly. However, there are occasions when families became numb when the conversation becomes too technical (Box F).

Box F The family was explained how the disease was behaving internally in detail, and how that is causing the complications, and why it was getting difficult each passing day.

The doctor added, ‘*Cancer has relapsed and has spread to the entire body. The bone of the forearm has got compressed due to the tumor’.*

The conversation from that point was very technical, went for approximately an hour, making information retention difficult.

The mother hearing all of these became very quiet and continued looking at the doctor without blinking her eyes.

#### Observation 5: Children may be passive participants, but they listen intently

**Figure 2.**
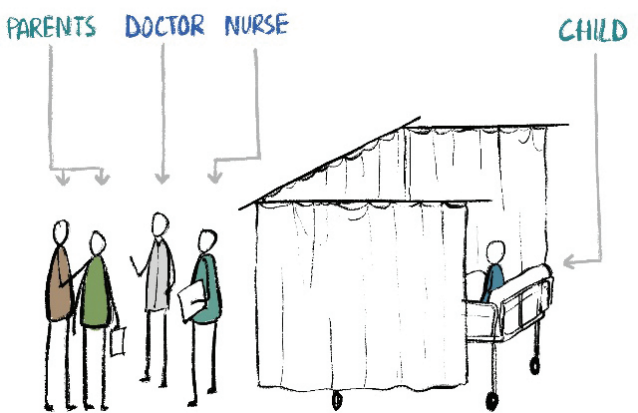
Children listening to adult conversations in paediatric wards.

In the children’s ward, the discussion about a child’s illness often happened at the foot-end of the bed with the curtains drawn around the child. The parents and professionals stand outside the curtains and may inadvertently forget about the possibility of the child overhearing them (Figure 2). One child engaged in sustaining this myth of ‘not knowing’. Until she was spoken to, she never indulged in any disease-related conversations with the adults. Once invited she asked relevant questions that made it evident that she knew all along.

#### Observation 6: The presence of members of the extended family may have a healing effect

In some of the conversations with the family, there was only one family member present. The entire onus of decision-making seemed to rest on one person. They also had to take the responsibility of conveying the information to other members of the family. Such situations could be difficult. It was observed that it was not unusual to have a member of the extended family be part of this conversation. This seemed to be of help during these difficult interactions. When parents struggled with absorbing the shock of the news, other family members could lead parts of the discussions. Therefore, it was observed that some doctors preferred sharing information with more than one individual member of the family when available. It helped them collectively overcome the shock and support one another. When one member could not continue the conversation, others intervened and continued the discussion (Box G). They believed in collecting as much information as possible to understand the situation and pull through.

Box G *After getting to know about the relapse, the patient’s father was in a state of shock. However, the uncle who accompanied them patted the patient on his head and provided comfort, and communicated with the doctor. The father and the child finally calmed down.*

#### Observation 7: Breaking bad news is stressful for clinicians before, during and after discussions

Before engaging in a potentially difficult conversation, doctors often discuss what to say and primed the team so that everyone was mentally prepared for the session. During such discussions, they often expressed their uneasiness and vocalised concerns for families they were planning to meet. Being able to empathise and at the same time not becoming too upset is not easy. In many of the sessions, the clinicians became emotional, either before (Box H), during (Box I) or after (Box J) the sessions.

Box H: **Emotional distress expressed by a clinician before meeting the family**

Dr. X said, ‘I am more scared because of the mother. His mother has been with him throughout his treatment. Probably she also has realized that the end has come. She would not leave the child even for a second. The only time she leaves him is when she has to go to the toilet. It would be very difficult for her to cope with the news. Very difficult. I am really worried about her. But we have to do what we have to do. We will go ahead and break this news’.

Box I: **Clinician becoming emotional during the discussion with a family**

After interacting with parents of a child who had failed the second line of treatment, one of the clinicians reflected, ‘When the father and the grandfather said that “no matter what, we will continue until the end, so that, later we know that we tried everything to save the child”, that made me cry. As a father I would do the same. I have a child of the same age. I cannot imagine how difficult it would be for me if the same thing was told to me’.

The doctor cried once more after this. He regretted breaking down in front of the patient’s family.

He said, ‘I shouldn't have cried. But it becomes so overwhelming when you see the helplessness and desperation in the family's eyes. Even we are helpless and our hands are tied. I wish we could save the child’.

Box J: **Emotions expressed by a clinician after completion of discussion with a family**

The brother of the patient had mentioned how patient B used to be the best student in his class. The teachers and relatives had extended them all sorts of help, both financially and academically. He had secured the first position in Physics Olympiad. After the session ended, the doctor who broke the news had tears in her eyes. Dr. A: ‘It is indeed quite unfortunate that he had to go through this’.

Breaking bad news is difficult for doctors, and they often express distress about this. Usually, they try to be honest, empathetic and sensitive. Surprisingly, sometimes parents could provide emotional support to the doctor who became upset while sharing the news of disease progression. Emotions expressed by the clinicians helped the families feel connected to the doctors. The people taking part in the conversation felt attuned to each other and the grave nature of the situation. Such bonding strengthened the trust and faith of parents in doctors, as was observed in one of the interviews (Box K).

Box K: “Sir, no, please don’t cry. You are God to me. You are truly God to me. You do not know how much trust we have in you. We know that you are trying. This time it will be luck’.

## Discussion

This study elicited the information-sharing experiences of professionals working in the field of paediatric oncology. The information and communication needs of children were reported to be different from adolescents and their parents. Professionals stressed the importance of ‘being able to listen and understand’ and ‘communicating a cohesive message to the family from all the team members’. Observation of communications episodes between parents and professionals highlighted the significance of ‘priming of children and parents before breaking bad news, ‘avoidance of premature reassurances’ and ‘involving members of the extended family when available’ during communications with children and their parents.

A key finding of the study was the need for the clinician to be attuned to the emotional state and the information needs of the parents before embarking on the task. In a recent study in north-eastern Iran, mothers of children with cancer preferred receiving any bad news regarding their child’s health from the doctor in charge of the child’s care in a compassionate way [20]. Observations made during our study also supported the value of priming parents before delivering bad news. Previous research carried out in India showed that children with cancer had variable degrees of understanding about their disease but were almost always excluded from the decision-making [21]. Observations made during our study revealed that children may listen to the communication between adults even if the communication was not directed at them. Our results showed that clinicians expressed that occasionally they did involve the children in the discussions after getting permission from parents, but majority of the observations supported the central role of discussion between parents and clinicians. Our study also found that information delivered very quickly may cause losing the purpose of the communication. This observation is supported by previous research in two paediatric hospitals in Tehran, Iran, which showed that an insensitive way of ‘dumping information’ without proper consideration of mothers’ mental preparedness and breaking bad news without enough priming could cause a significant amount of stress [22]. Sharing too much information too quickly could be psychologically traumatic for the parent, and previous research from Boston, USA, showed that many parents of children with cancer, even in developed countries, preferred a tempered and gradual approach [23]. One consistent observation made in our study was the effectiveness of allowing some time to help parents process the information between episodes of communications. This has been found in other settings, e.g., in the USA with parental responses to hearing their child had neurofibromatosis and the experiences of a Canadian paediatrician speaking with parents of children who are dying [24, 25]. A study on parents of children with cancer in the USA [23] showed that communication had several different purposes, such as building a relationship, exchanging information, enabling self-management of the family, responding to emotions, managing uncertainty, assisting in decision-making, providing validation and supporting hope.

A study conducted in the Netherlands reported on parental views on receiving bad news on their child’s life-limiting condition [26] and highlighted the concerns raised by parents related to lack of timely communication, physician’s failure to voice uncertainties, parental concerns around breaking bad news to children and difficulties arising out of misunderstanding medical terminologies. Health professionals who participated in our study also reported almost universal information needs of parents of children with cancer with some inter-individual differences. We report that educated parents may be more empowered to ask questions. It is crucial to engage parents of children with cancer in a culturally acceptable way. In doing so, one needs to assess how much information is welcomed by the family as there are cultural variations in medical information seeking and sharing between clinicians and the patient [27]. Recent research from Guatemala showed that at the time of diagnosis of children’s cancer, parental perceptions may be routed in prevalent cultural nihilism around cancer, which may change over time to a more optimistic outlook following engagement with the health service providers [28]. A systematic review published from Taiwan that included studies published in Chinese had reported on the emotive aspects of information sharing and the importance of regular communication between family members of children with cancer and health professionals [29]. A comprehensive systematic review on communication during childhood cancer that included studies from high-income countries, such as USA, UK and Germany, and also LMICs, such as China, Iran, Puerto Rico and Tanzania, reported the value of open and honest communication in paediatric oncology [8].

Previous research in Turkey with children aged 7–12 years undergoing cancer treatment viewed a doctor or a nurse positively if they communicated with them as well as their parents [30]. The presence of a multidisciplinary team [22] may be a helpful strategy during communication as was reported by a study conducted on children with cancer from Iran. One study from Iran reported on the additional psychological stress on mothers of children with cancer who had to convey the bad news to other family members and the authors advocated involving the extended family in clinical communications [31]. Another study also from Iran showed that health professionals valued communicating with patients and their extended families and perceived that this helped in emotional bonding with the patient [32]. Our study emphasised the importance of the presence of extended family members and this is likely to be culturally embedded. A previous study on adult cancer patients with advanced disease conducted in Canada showed that family members should be present with patients to deal with the bad news [33]. Our study emphasised the importance of having more than one family member while communicating bad news. We observed that different family members may be able to support each other. This is in line with the findings reported from the USA by a review published on communications in paediatric oncology. This review summarised the value of involving the extended family so that they help each other to adjust to their new roles, manage their emotions better, communicate with the patient appropriately and even cope with the aftermath of the death of a child if that happens [34].

Our study showed that breaking bad news can be stressful for clinicians as well. This is in line with a recently published review on professional burnout in paediatric haematology and oncology from the United States [35]. Breaking bad news becomes more complex for beginners, who have to learn the technique only through observation of experienced physicians [36]. Physicians may develop a profound attachment with their patients and find it challenging to deal with their own emotions, which may be overwhelming after a patient’s death [37]. Death can have grave consequences on paediatric oncologists, causing grief, helplessness, sadness and feelings of failure [37]. However, when handled with empathy and humility, end-of-life conversations build a connection between individuals that may be deeply meaningful to the participants and the impact is likely to be much beyond the time of loss [38]. Our research elicited some of the nuances around end-of-life communication preferences as expressed by paediatric oncology professionals. Several of our interviews suggested that adolescent cancer patients may choose to talk about their death. Research on end-of-life conversations from the USA showed that most of it happened in the last few days of the young person remaining alive. This often leaves very little time to address any unfulfilled desire or achieve closure on any specific issue. This seems to be reflected in our study as well. One of the recently published guidelines on end-of-life communication practices published by authors from Australia and USA emphasised that adolescents and young adults may have strong preferences on the matter of their end of life and often choose to be at home, be pain-free and express their wishes to their loved ones [41].

There are several strengths of the study. The study adhered to the COREC guidelines for qualitative research [42]. The interviewers were not part of the paediatric oncology team and consisted of a sociologist and clinical psychologist who put the respondents at ease. The paediatric oncology professionals included in the study had varied backgrounds and were representative of multidisciplinary teams looking after children with cancer. They could thus provide their unique and different perspectives. The information obtained from the interviews complemented the observation of real-life communication in the outpatient department, inpatient wards, emergency department and day care services for children. A limitation of the study was that it was conducted in a single not-for-profit cancer centre in eastern India and thus may not be representative of all settings across the country.

### Implications for clinicians

The present research highlights the importance of communication in paediatric oncology. It is vital for paediatric oncology healthcare teams to actively think about ways to deal with information sharing with paediatric patients, their parents and families. Our research suggests that clinicians should be supported to develop the skills in handling these difficult conversations. The clinical consultation and communication habits of paediatric oncology healthcare professionals are best embedded in their routine practice of reflection about communication with patients and their parents. Learning to communicate with children and their parents should be part of the personal development plan of individual staff working in paediatric oncology. There may be psychological strain on professionals working with children with cancer who are sick or dying with cancer. The emotional health of paediatric oncology healthcare workers needs to be prioritised by team leaders while mentoring juniors and colleagues.

### Implications for policymakers

**Figure 3.**
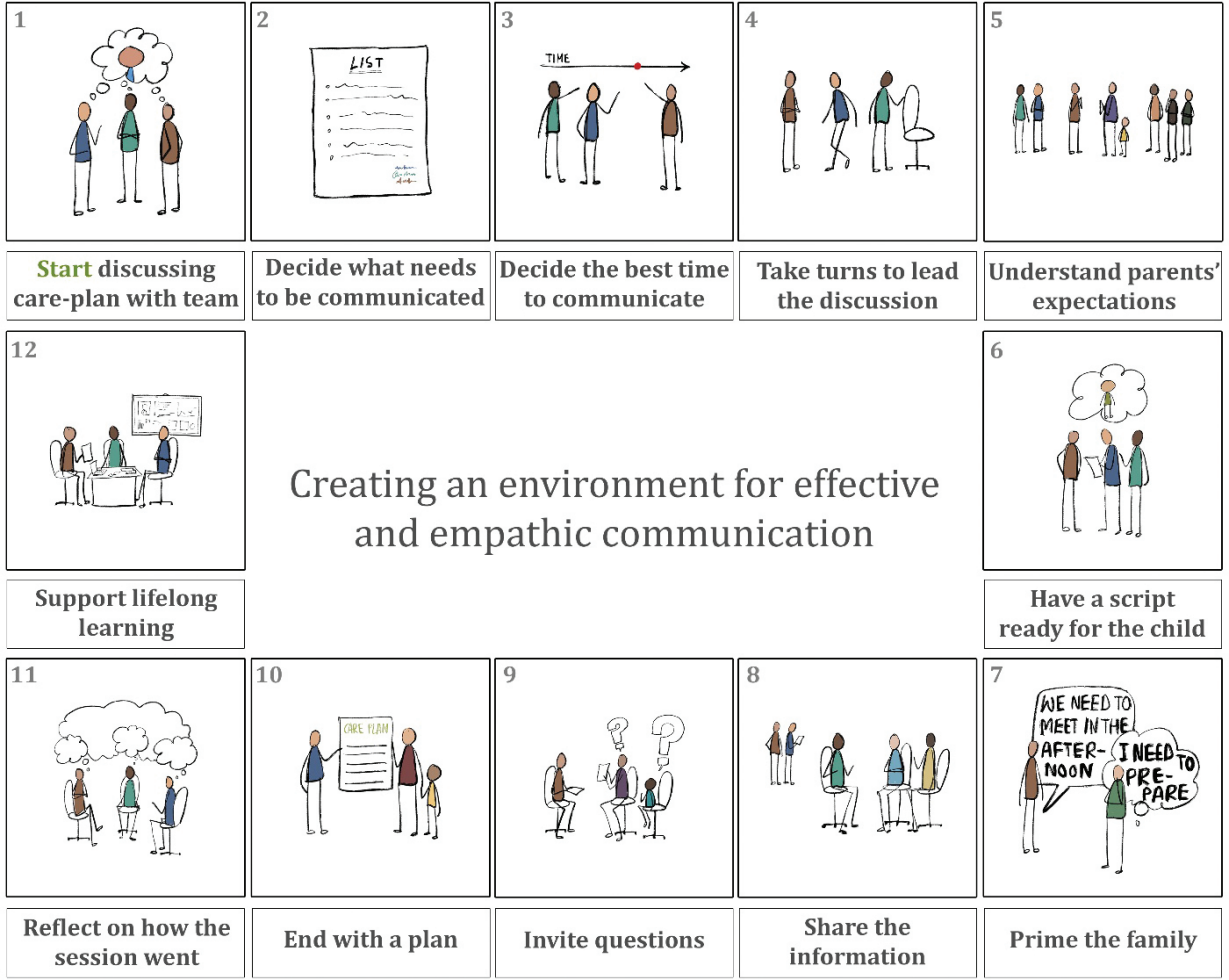
Creating an environment for effective and empathic communication in paediatric oncology.

The medical planners should try to create an environment that encourages better communication between clinicians, patients, family members and between different team members. Adequate staffing levels also make staff less rushed while answering the queries of the patients and parents. Availability of a comfortable sitting place suitable for parent–professional meetings will likely encourage more frequent communications. Institutions should provide opportunities for clinicians to develop their communication skills. Having a work culture that encourages staff to reflect on communication practices and supporting staff to learn the soft skills of communicating with children, young people and their parents go a long way to make a unit provide a stable foundation of care. We propose a model for creating an environment for developing empathic and effective communication in paediatric oncology (Figure 3).

### Future research implications

The present research sheds light on the communication preferences of professionals working in a paediatric oncology setting in an LMIC. We suggest future research focus on developing and testing communication skills training programmes for paediatric oncology teams working in LMICs. Typically, most paediatric oncology teams in LMICs work under the high pressure of managing many patients and with fewer resources than in the developed countries. The communication skills training methods need to be attuned to the needs of the unit where it is to be delivered. At the same time, communication skills training programmes should have a robust evidence base to devote scarce man-hours to participate in this training and make it worthwhile.

## Conclusion

The study highlights the preferences of paediatric oncology professionals on information-sharing practices in an LMIC. Five broad themes highlighted the nuances of communication with children, adolescents and their parents, alongside professional preferences of communication in paediatric oncology. The study also showed that priming the recipient of information helps in the communication process. Whenever appropriate, clinicians should be ready to communicate with children and adolescents directly to engage them in the treatment process. Clinicians may consider inviting additional family members during difficult conversations. We believe this work could be the basis for developing further training resources in the future. The information needs of parents and children with cancer may be different. Data suggest that future communication skills training for paediatric oncology clinicians should include training on the specific information needs of children, adolescents and parents. A private setting with protected time to communicate with the patient and his or her family is preferred. Paediatric oncology units should provide the foundation for compassionate communication between children, their parents and clinicians.

## Conflicts of interest

The authors declare that they do not have any conflicts of interest.

## Figures and Tables

**Table 1.** Paediatric oncology professionals who participated in the study.

	*n* = 14	Percentage
**Professional interviewed**		
Consultant paediatric oncologist	2	14.3
Trainee doctor in paediatric oncology	3	21.4
Nursing	2	14.3
Psychologist/Counsellor	2	14.3
Specialist social worker	2	14.3
Survivor counsellor cum social worker	1	7.1
Clinical nutritionist	1	7.1
Clinical pharmacist	1	7.1
**Age**		
20–29	3	21.4
30–39	6	42.8
40–49	4	28.6
50–59	1	7.1
**Gender**		
Male	5	35.7
Female	9	64.3
	Median	IQR (Range)
Number of years in paediatric oncology (in years)	4.35	IQR 2.5–8, (Range 1–20)

**Direct observation of interactions**	***n* = 10**	**Percentage**
**Timing of observation**		
At diagnosis	2	20
During curative treatment	2	20
At relapse	2	20
At the transition from curative to palliative treatment	1	10
During palliative treatment	3	30
		
**Observation site**		
Outpatient department	6	
Emergency department	1	
Day care	1	
Counselling room in the ward	2	
